# Health-related quality of life and social support among women treated for abortion complications in western Uganda

**DOI:** 10.1186/1477-7525-11-118

**Published:** 2013-07-15

**Authors:** Solomon J Lubinga, Gillian A Levine, Alisa M Jenny, Joseph Ngonzi, Peter Mukasa-Kivunike, Andy Stergachis, Joseph B Babigumira

**Affiliations:** 1 Department of Pharmacy, Pharmaceutical Outcomes Research and Policy Program, University of Washington, Seattle, WA, USA; 2 Department of Global Health, Global Medicines Program, University of Washington, Seattle, WA, USA; 3 Department of Obstetrics and Gynecology, Mbarara University of Science and Technology, Mbarara, Uganda; 4 EngenderHealth Fistula Care Project, Kampala, Uganda; 5 Department of Epidemiology, University of Washington, Seattle, WA, USA

**Keywords:** Health-related quality of life, EQ-5D, Abortion complications, Social support

## Abstract

**Background:**

While the impact of abortion complications on clinical outcomes and healthcare costs has been reported, we found no reports of their impact on Health-Related Quality of Life (HRQoL), nor the role of social support in moderating such outcomes. In this study, we performed an assessment of the relationship between abortion complications, HRQoL and social support among women in Uganda.

**Methods:**

We interviewed women who were discharged after treatment for abortion complications and, as a comparison, women visiting a regional referral hospital for routine obstetric care. We administered the EuroQol instrument and the Social Support Questionnaire Short-Form, and collected demographic and socioeconomic data. We performed descriptive analyses using t-tests, Wilcoxon rank-sum tests and chi-square tests, and multivariable linear regressions with interaction effects to examine the associations between abortion complications, EQ-5D utility scores and social support.

**Results:**

Our study included 139 women (70 with abortion complications, and 69 receiving routine obstetric care). In four out of the 5 dimensions of the EQ-5D, a larger proportion of women with abortion complications reported “*some or severe*” problems than women receiving routine obstetric care (self-care: 42% v 24%, p=0.033; usual activities: 49% v 16%, p<0.001; pain/discomfort: 68% v 25%, p<0.001; and anxiety/depression: 60% v 22%, p<0.001). After adjusting for age, social support, wealth tertile, employment status, marital status, and HIV status, women with abortion complications had a 0.12 (95% CI: 0.07, 0.18, p < 0.001) lower mean EQ-5D utility score than those receiving routine obstetric care. An analysis of the modifying effect of social support showed that a one-unit higher average number of people providing social support was associated with larger mean difference in EQ-5D utility score when comparing the two groups, while a one unit higher average satisfaction score with social support was associated with smaller mean differences in EQ-5D utility score.

**Conclusions:**

Our study suggests that abortion complications are associated with diminished HRQoL and the magnitude of the association depends on social support. However, the mediating role of social support in a setting of social and legal proscriptions to induced abortion is complex.

## Introduction

In Uganda, where abortion is illegal except to save the life of the mother, illegally-induced abortions are often carried out in unsafe conditions, by unskilled providers. Unsafe abortions and resultant complications represent a significant burden to women and the healthcare system. Singh et al. [1] estimated that one in five pregnancies result in an abortion, for an estimated 297,000 induced abortions annually. Of these, at least 85,000 result in complications necessitating treatment. Recent studies in Uganda have documented the substantial economic costs associated with unsafe abortions and resulting complications. Babigumira et al. [2], using a decision-analytic model to represent the consequences of an induced abortion, estimated an annual per-patient societal cost of $171, translating into $64 million in costs per year for the country. In an empirical analysis, Vlassoff et al. [3] found that the annual cost of treating abortion complications was approximately $131 per case and $13.9 million per year in Uganda, and an estimated $20.8 million per year that would be needed to satisfy all demand for management of abortion complications in the country. Though cost estimates differ—likely as a consequence of the different methodologies and perspectives adopted by the studies—both highlight the substantial negative economic impact of induced abortions on the health care system and society.

In addition to economic impact, the clinical and health consequences of illegally-induced and often unsafe abortions have been described elsewhere [4,5]. However, much less is known about the impact on patient reported outcomes, specifically Health-Related Quality of Life (HRQoL). Westhoff et al. [6], describe improvements in quality of life (measured using the European Organization for Research and Treatment of Cancer Quality of Life Questionnaire) comparing medical to surgical abortions in a setting where abortions are legal, are carried out in a specialist setting, and where both procedures are accompanied by a low risk of complications. No studies were identified that reported on the relationship between complications resulting from illegally-induced abortions and HRQoL, nor the role of social support in moderating this relationship. Social support is particularly important in this setting in light of the significant moral and religious proscriptions to abortions, as well as the attendant social stigma. Lack of social support is commonly cited as a reason for seeking an induced abortion [7], and could conceivably affect HRQoL, even in the absence of abortion or abortion complications. Yet when available, social support is associated with improved psychological outcomes following an abortion, and following treatment for abortion complications [8,9]. Differences in HRQoL may vary depending on availability of and satisfaction with social support.

In this study, we sought to examine the relationship between abortion complications, HRQoL and social support. We hypothesized that, after adjusting for socioeconomic status (SES) indicators, co-morbid conditions and social support, women who have experienced abortion complications would have a significantly diminished HRQoL. We also hypothesized that the magnitude of the reduction in HRQoL would vary, depending on the reported availability of, and/or level of satisfaction with social support.

## Methods

### Subjects and settings

The study was conducted at Mbarara Regional Referral Hospital, a 300-bed hospital that serves as the referral center for western Uganda and academic teaching hospital for the Faculty of Medicine of Mbarara University of Science and Technology. This baseline analysis of health-related quality of life and social support utilizes data from a prospective cohort study to evaluate the impact of abortion complications on health and economic outcomes of women in Uganda [2,10]. Between December 2009 and October 2010, we enrolled women at discharge who had been admitted to the Obstetric and Gynecology unit for treatment of complications resulting from induced abortions. As a comparison, we enrolled women visiting the same hospital unit, during the same time period, for routine obstetric visits. Women were eligible to participate if they were above 18 years old, and consented to the interview and follow up.

### Procedures

We used the EuroQol instrument to measure HRQoL [11]. This is a standardized instrument that measures health status in 5 dimensions—mobility, self-care, usual activity, pain and anxiety/depression (EQ-5D). For each dimension, the respondent selects one of 3 levels—no problems, some problems and severe problems—giving a total of 243 possible health states. These are then combined into a single utility score. The instrument also measures a respondent’s self-rated health on a linear, visual analogue scale (EQ-VAS). A single EQ-5D utility score for our participants was computed using a scoring algorithm based on a general population survey in Zimbabwe [12].

Social support was measured using the Social Support Questionnaire (SSQ), Short Form [13], a standardized six-item, two-part instrument that measures a respondent’s social support on two scales: a social support questionnaire number (SSQN) scale and a social support questionnaire satisfaction (SSQS) scale. A SSQN score is computed by averaging the number of people reported on items that ask about the people in a respondent’s environment who provide different forms of help and support. A SSQS score is computed by averaging the responses to items that rate a respondent’s satisfaction with the support that they receive using a Likert-scale. Higher total scores on each of the scales indicate greater social support. The SSQ has demonstrated satisfactory test-retest reliability and internal consistency [13].

We collected demographic data including age, marital status and highest level of education, and data on ownership of various durable goods and housing characteristics and medical and obstetric history including number of pregnancies, number of children, self-reported HIV status and other chronic medical conditions. Among women who had experienced abortion complications, we collected data on circumstances surrounding the abortion—contraceptive use and whether the pregnancy was intended, duration of pregnancy prior to the first abortion attempt and the method used at the first abortion attempt.

A trained research assistant administered the instruments and interviewed eligible women after obtaining written informed consent. For the abortion complications group, we consecutively enrolled consenting women prior to discharge following treatment for abortions complications. For the routine obstetric care group, we also consecutively enrolled eligible consenting women following their visit to the Obstetrician. Our primary outcome measure was the mean EQ-5D utility score. A post-hoc power analysis showed that, with 70 patients in the abortions complications group and 69 in the routine obstetric care group, we had approximately 84% power to detect a clinically meaningful difference of 0.10 in EQ-5D utility score between the two groups at 5% significance level assuming a standard deviation of the mean EQ-5D utility score of 0.2 in both groups.

### Statistical analyses

The data were entered into a database in Microsoft Excel 2010 and exported to STATA 12 (Stata Corporation, Texas, USA) for analysis. First, we used the data on ownership of durable goods and housing characteristics to construct a wealth index score by Principal Component Analysis using the first principal component [14]. This score was then re-categorized into tertiles. Participant characteristics were summarized, age as mean and standard deviations (SD) and number of children as median and Interquartile Range (IQR). Categorical variables were summarized as the number and proportion in each category. We conducted bivariate analyses to compare participant characteristics between the two patient groups. Age was compared using an independent samples t-test while the number of children was compared using the Wilcoxon rank sum test. Bivariate analyses of categorical variables were conducted using chi-square tests.

We compared the mean SSQN and SSQS scores, and EQ-5D utility and EQ-VAS scores between the two participant groups using independent samples t-tests and used Pearson’s correlation to determine whether there was correlation between EQ-5D utility score and the social support measures. Few women reported severe problems in any of the domains of the EQ-5D. We combined the categories, “*some problems*” and “*severe problems*” for each of the dimensions, creating a binary response: “*no problems*” versus “*some or severe problems*”, and used chi-square tests to compare the proportion of responses in each category of the EQ-5D domains between the two participant groups.

We used multivariable linear regression models to examine associations between HRQoL and abortion complications, adjusting for the pre-hypothesized confounders: age, social support, number of children, self-reported HIV status and indicators of socioeconomic status (SES), measured using wealth index, marital status and employment status. To examine whether perceived social support modified the association between abortion complications and HRQoL, we reconstructed the multivariable linear regression model adding two interaction terms, the first between the main explanatory variable and the SSQN score and the second, between the main explanatory variable and the SSQS score. The dependent variable in the regression models was the EQ-5D utility score and the main explanatory variable was participant group: abortion complications or routine obstetric care. Post-hoc partial F-tests and multiple partial F-tests were used to test the null hypothesis that the interaction terms individually and together were equal to zero i.e., that the difference in EQ-5D utility scores between the two groups did not depend on SSQN and SSQS scores.

### Ethics statement

The study was approved by the University of Washington Institutional Review Board, the Institutional Review Committee of Mbarara University of Science and Technology, and the Uganda National Council of Science and Technology. All participants provided written informed consent.

## Results

### Participant characteristics

We enrolled 139 women, 70 hospitalized with abortion complications, and 69 receiving routine obstetric care. Baseline characteristics are shown in Table 1. The overall mean (SD) age was 28.8 (5.7), with no difference between the abortion complications and routine obstetric visit group. There were neither differences in educational attainment, nor employment status between groups. The groups differed in the number of children, marital status, self reported HIV status and wealth index summary.

**Table 1:** Participant characteristics, Mbarara Regional Referral Hospital, December 2009 – October 2010

**Variable**	**All participants N = 139**	**Routine obstetric care, N = 69**	**Abortion complication, N = 70**	**p-value**
Age (years), mean (SD)	28.8 (5.7)	29.0 (5.0)	28.7 (6.3)	0.790^†^
Number of children, median (IQR)	3 (1, 4)	3 (2, 4)	2 (0, 4)	0.002^§^
0	20 (14.3)	2 (2.9)	18 (25.7)	0.001^‡^
1-3	68 (48.3)	36 (52.2)	32 (45.7)	
4-6	32 (23.0)	22 (31.9)	10 (14.3)	
>6	19 (13.7)	9 (13.0)	10 (14.3)	
Highest education level				
No schooling	21 (15.3)	12 (17.9)	9 (12.9)	0.252^‡^
Part primary	29 (21.2)	14 (20.9)	15 (21.4)	
Completed primary	24 (17.5)	7 (10.5)	17 (24.3)	
Part secondary	27 (19.7)	13 (19.4)	14 (20.0)	
Completed secondary	18 (13.1)	12 (17.9)	6 (8.6)	
More than secondary	18 (13.1)	9 (13.4)	9 (13.8)	
Marital status				
Currently married	95 (68.4)	56 (81.2)	39 (55.7)	0.011^‡^
Never married	31 (22.3)	8 (11.6)	23 (32.9)	
Separated/divorced	11 (7.9)	4 (5.8)	7 (10.0)	
Widowed	2 (1.4)	1 (1.5)	1 (1.4)	
Self-reported HIV status				
Negative	71 (51.8)	44 (63.8)	27 (38.6)	0.004^‡^
Positive	18 (13.1)	10 (14.5)	8 (11.8)	
Don’t know	48 (35.0)	15 (21.7)	33 (47.1)	
Employment status				
Not working/Subsistence worker	96 (69.6)	49 (71.0)	46 (68.1)	0.784^‡^
For money, Part-time	23 (16.7)	10 (14.5)	13 (18.8)	
For money, Fulltime	19 (13.8)	10 (14.5)	9 (13.0)	
Socioeconomic Index summary				
Lowest tertile	41 (33.3)	21 (39.6)	20 (28.6)	0.012^‡^
Middle tertile	41 (33.3)	10 (18.9)	31 (44.3)	
Highest tertile	41 (33.3)	22 (41.5)	19 (27.1)	

^†^ independent samples t-test.

^‡^ chi-square test.

^§^ Wilcoxon rank-sum test.

Among the abortion complications group, 48 women (68.57%) reportedly used contraceptives prior to the pregnancy, and of these, 26 women (56.52%) used the commonly available pill-plan®. Sixty-three women (90%) reported that the pregnancy was unintended. The median (IQR) pregnancy duration prior to first abortion attempt was 12 (8, 16) weeks. The median (IQR) number of abortion attempts was 1 (1, 2). A variety of methods were used at the first abortion attempt. The most common methods were herbs or other product taken orally (41.43%), insertion of object into the vagina (21.43%) and surgical procedure (12.86%).

### Health-Related Quality of Life

**Figure 1:**
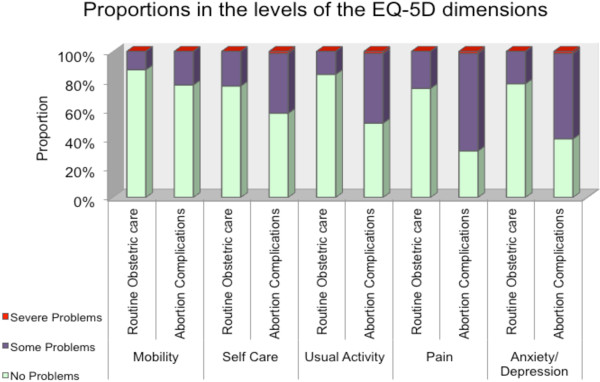
Proportions reporting severe problems, some problems or no problems in the dimensions of the EQ-5D.

A larger proportion of women with abortion complications reported “*some or severe problems*” than did women receiving routine obstetric care in four of the five dimensions of the EQ-5D (Figure 1) including self-care (42% v 24%, p = 0.033), usual activities (49% v 16%, p < 0.001), pain/discomfort (68% v 25%, p < 0.001), and anxiety/depression (60% v 22%, p<0.001); the difference was not significant for mobility (23% v 13%, p = 0.126).

**Figure 2:**
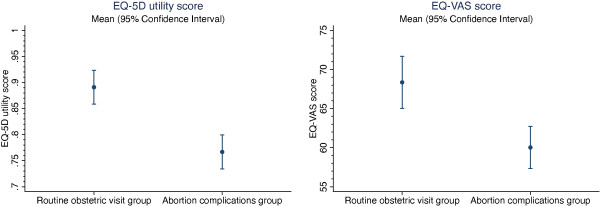
Comparison of EQ-5D utility and EQ-VAS scores, abortion complications group versus routine obstetric care group.

Women with abortion complications had a significantly lower mean EQ-5D utility score (0.77 v 0.89, p < 0.001) and EQ-VAS score (60 v 68, p < 0.001) than women visiting the hospital for routine obstetric care (Figure 2).

### Social support

Neither the mean SSQN scores (1.50 v 1.67, p = 0.278) nor SSQS scores (4.59 v 4.87, p = 0.247) differed significantly between women in the abortion complications group and the routine obstetric care group. There were neither correlations between EQ-5D utility score and SSQN for either group (abortion complications group: ρ = −0.18, p = 0.153; routine obstetric care group: ρ = 0.11, p = 0.384) nor between EQ-5D utility score and SSQS (abortion complications group: ρ = 0.05, p = 0.71; routine obstetric care group: ρ = 0.08, p = 0.555).

### Associations between HRQoL, social support and abortion complications

In a multivariable linear regression model adjusting for age, social support, number of children, marital status, self-reported HIV status, employment status, and wealth tertile (Table 2: model 1), women with abortion complications had a lower mean EQ-5D utility score than those receiving routine obstetric care (adjusted difference = 0.12, 95% CI: 0.07, 0.18, p < 0.001).

**Table 2:** Multivariable linear regression analyses of the association between abortion complications and EQ-5D utility score

**Variable**	**Model 1: no interaction terms**			**Model 2: with interaction terms**		
	**Difference in mean EQ-5D utility Score **^†,*^	**95% CI**	**p-value**	**Difference in mean EQ-5D utility Score **^†,**^	**95% CI**	**p-value**
Abortion Complications (versus Routine Obstetric Care)	−0.12	−0.18, -0.07	<0.001	−0.25	−0.47, -0.02	0.031
Age (years)	0.00	−0.01, 0.01	0.620	0.00	−0.01, 0.01	0.705
Social Support Number Score	0.02	−0.02, 0.05	0.389	0.07	0.01, 0.12	0.017
Social Support Satisfaction Score	0.03	0.00, 0.05	0.026	0.06	0.02, 0.10	0.007
Number of Children	0.00	−0.02, 0.01	0.687	−0.00	−0.02, 0.02	0.950
Marital status (versus Currently married)						
Never married	0.00	−0.06, 0.07	0.919	0.01	−0.05, 0.08	0.735
Separated/divorced	−0.09	−0.17, 0.00	0.053	−0.01	−0.19, -0.10	0.029
Widowed	0.15	−0.12, 0.41	0.272	0.13	−0.13, 0.39	0.313
Self-reported HIV status (versus Negative)						
Positive	−0.03	−0.11, 0.05	0.501	−0.02	−0.10, 0.06	0.588
Don’t know	−0.02	−0.08, 0.04	0.464	−0.02	−0.08, 0.04	0.511
Employment status (versus Not working or Subsistence worker)						
For money, Part-time	−0.11	−0.18, -0.04	0.004	−0.11	−0.18, -0.03	0.005
For money, Fulltime	−0.05	−0.13, 0.03	0.194	−0.04	−0.12, 0.04	0.288
Socioeconomic Index summary (versus Lowest tertile)						
Middle tertile	−0.06	−0.13, 0.00	0.048	−0.05	−0.11, 0.01	0.118
Highest tertile	0.00	−0.06, 0.06	0.980	−0.00	−0.06, 0.06	0.981
Abortion Complications * SSQN score				−0.09	−0.15, -0.05	0.018
Abortion Complications * SSQS score				0.05	0.00, 0.10	0.043
	F_(14, 91)_ = 3.68, p = 0.0001			F_(14, 89)_ = 3.81, p < 0.001		

^†^ the difference in mean EQ-5D utility score associated with a 1 unit higher value of the independent variable (continuous variables) or when comparing with the reference group (categorical variables).

^*^ adjusted for all other variables shown.

^**^ adjusted for all other variables shown and includes interactions between Abortion Complications and Social Support Questionnaire Number (SSQN) score and Abortion Complications and Social Support Questionnaire Satisfaction (SSQS) score.

In a model to assess the interaction between abortion complications and social support (Table 2: model 2), partial F-tests show that the coefficients for the interaction terms are significantly different from zero (abortion complications * SSQN score, coefficient = −0.085, F _(1, 89)_ = 5.81, p = 0.018 and abortion complications * SSQS score, coefficient = 0.052, F_(1, 89)_ = 4.21, p = 0.043). A multiple partial F-test also shows that interaction terms, jointly, are significant additions to the model (F_(2, 89)_ = 3.37, p = 0.038), suggesting that the difference in mean EQ-5D utility score between the two groups depends on both the SSQN and SSQS score. At the mean SSQN and SSQS score, the abortion complications group had 0.13 (95% CI: 0.08, 0.19, p < 0.001) lower mean EQ-5D utility score than the routine obstetric visit group. Comparing women with abortion complications and those visiting the hospital for routine obstetric care, one-unit higher average SSQN score was associated with progressively larger mean differences in EQ-5D utility score, holding SSQS score constant; whereas a one-unit higher average SSQS score was associated with progressively smaller mean differences in EQ-5D utility score, holding SSQN score constant (Table 3).

**Table 3:** Estimated difference (95% CI) in mean EQ-5D utility score for different SSQN and SSQS combinations

		**Social support questionnaire satisfaction (SSQS) score**					
		1	2	3	4	5	6
Social Support Questionnaire Number (SSQN) Score	1	−0.28	−0.23	−0.17	−0.12	−0.07	−0.02
		(−0.47, -0.09)	(−0.37, -0.08)	(−0.27, -0.07)	(−0.19, -0.05)	(−0.14, 0.00)	(−0.12, 0.08)
	2	−0.36	−0.31	−0.26	−0.21	−0.15	−0.10
		(−0.58, -0.14)	(−0.48, -0.14)	(−0.38, -0.13)	(−0.29, -0.12)	(−0.21, -0.09)	(−0.17, -0.03)
	3	−0.45	−0.40	−0.34	0.29	0.24	−0.19
		(−0.72, -0.18)	(−0.62, -0.17)	(−0.52, -0.17)	(−0.43, -0.15)	(−0.35, -0.13)	(−0.29, -0.09)
	4	−0.53	−0.48	−0.43	−0.38	−0.32	−0.27
		(−0.85, -0.21)	(−0.76, -0.20)	(−0.67, -0.19)	(−0.58, -0.17)	(−0.50, -0.15)	(−0.43, -0.11)

## Discussion

In this study comparing Health-Related Quality of Life (HRQoL) between women discharged following treatment for abortion complications and those visiting the hospital for routine obstetric care, we found that abortion complications were associated with a significantly diminished HRQoL, and that this association depended on social support. This is the first study, in a resource-limited setting that attempts to describe the relationship between abortion complications, HRQoL and social support.

Women with abortion complications had lower EQ-5D utility scores and EQ-VAS scores compared to women seeking routine obstetric care, a marker of significantly diminished HRQoL. We also found that higher proportions of women with abortion complications reported either some or severe problems with self-care, usual activity, pain/discomfort and anxiety/depression compared to the women visiting the hospital for routine obstetric care. The largest differences were observed in the pain/discomfort, usual activity and anxiety/depression dimensions, suggesting that these are the main drivers of poorer quality of life. Previous research has shown that undergoing an abortion may be associated with adverse psychological outcomes, including depression, suicide-related admissions, feelings of regret, low self-esteem, substance abuse, and deliberate self-harm [15]. Therefore, one can surmise that complications following an illegally-induced abortion may be associated with more severe sequelae. The impact of abortion complications on physical health has also been described. Women who do not die following an unsafe abortion are at risk of short-term consequences including severe bleeding, vaginal and abdominal injury which often result in hospitalization [4,5]. These sequelae are associated with impairment in the performance of one’s usual activities, self-care, mobility and pain and discomfort.

In our study, the adverse effects of abortion complications on HRQoL were maintained even after adjusting for potential confounders including age, social support, HIV status and socioeconomic indicators, demonstrated by the significant difference in mean EQ-5D utility score in adjusted models. Social support measures were included in the regression models first as a potential confounder, because lack of social support, especially from immediate family, has been cited as a reason why a woman may seek an abortion [7]. However, lack of social support may also independently be associated with poor health related quality of life. Studies in settings where abortion is legal have shown that social support may lead to improved psychological outcomes following an induced abortion [8,9]. We therefore extended a similar argument to the treatment of abortion complications, assuming that social support would be protective in the association between abortion complications and HRQoL. On average, women in each group reported similar levels of social support. However, we found that the impact of abortion complications on HRQoL was dependent on the number of people who a respondent listed as providing support (SSQN score) and the level of satisfaction with the support received (SSQS score). Our model suggests a paradoxical effect of SSQN score on the difference in HRQoL between our study groups where a one unit higher average number of people providing social support, was associated with larger mean differences in HRQoL, when comparing women with abortion complications and those visiting the hospital for routine obstetric care. This implies that women treated for abortion complications have worse HRQoL, the larger the average number of people providing social support, when compared to those receiving routine obstetric care. One potential explanation for this finding may result from an inherent desire for confidentiality in this setting of substantial anti-abortion stigma. Women who experience abortion complications might prefer to confide in fewer people, whereas in the comparison group (the routine obstetric participants, for whom there was likely no stigma), having more people in whom to confide is associated with higher utility. On the other hand, the more satisfied a woman was with the support she received, the smaller the difference between the groups in utility scores, suggesting a tendency towards at least similar HRQoL. HRQoL may be better if women experiencing abortion complications were highly satisfied with the support offered by those few people in whom they confide. We find these results plausible in a setting of high religious morality, legal proscriptions, and substantial anti-abortion stigma, all of which might prevent women from wanting to confide in more people.

There are several limitations of this study. First, this is a single-center, cross-sectional study of limited numbers, in one region of Uganda. Therefore, interpretations beyond this setting should be made cautiously. There may be unmeasured cultural factors that might bear on the generalizability of these findings, particularly with respect to the social support structures in place. Although it may be a dynamic measure, we did not investigate the changes in social support that women may experience along the course of pregnancy, undergoing an abortion, during admission or post-discharge for abortion complications, nor in the long term. It is possible that given this setting in which abortion is illegal and carries significant social and religious stigma, the perceptions of support available may change and these changes might be important in elucidating the social mechanisms for coping with an induced abortion and its complications. There are also limitations inherent in the use of EQ-5D and the SSQ Short Form in this setting. First, neither instrument has been validated in this region and no official version was available in the local language for the region (Runyankore). We used an algorithm based on Zimbabwean general population data to compute the EQ-5D utility score, and though we expect this is the best available population equivalent, the groups are likely somewhat different. Finally, this study reports specifically short-term HRQoL.

## Conclusions

This study adds to the body of evidence highlighting the significant adverse effects of abortion complications on health outcomes [4,5] by reporting the negative impact of abortion complications on HRQoL, and the mediating role of social support in a resource-limited setting. The study suggests a potential benefit of appropriate social support. However, the type and structure of social support may be complex in a setting with legal, moral and religious proscriptions to induced abortions. Improving access to strategies that prevent unsafe abortions, such as better access to sex education, increased access to contraceptives [10], and safe legal abortion services should remain a priority area in policy-making. Research into strategies to prevent unsafe abortions and appropriately manage abortion complications should consider HRQoL as an important outcome.

## Competing interests

The authors declare that they have no competing interests.

## Authors’ contributions

SJL participated in data entry, designed and performed statistical analyses and drafted the manuscript. GAL participated in data cleaning, interpretation of results and drafting/revising of the manuscript. AJ participated in manuscript revision. JN and PMK collected the data and participated in revising the manuscript. AS provided comments on designing the analysis and participated in revising the manuscript. JBB designed the study, participated in data collection, statistical analyses and drafting/revising the manuscript. All authors read and approved the final manuscript.
